# An integrated strategy for reducing anastomotic leakage in patients undergoing McKeown esophagectomy

**DOI:** 10.1016/j.heliyon.2024.e26430

**Published:** 2024-02-15

**Authors:** Yan Zhang, Junya Wang, Shuang Ren, Jia Jiao, Zheng Ding, Hang Yang, Dabo Pan, Jindong Li, Guoqing Zhang, Xiangnan Li, Song Zhao

**Affiliations:** a Department of Thoracic Surgery and Lung Transplantation, First Affiliated Hospital of Zhengzhou University, Zhengzhou, 450052, Henan Province, China; b Department of Oncology, Second Affiliated Hospital of Zhengzhou University, Zhengzhou, 450052, Henan Province, China

**Keywords:** Esophagectomy, Anastomotic leakage, Blood supply, Reconstruction route, Gastric tube dilation

## Abstract

**Objective:**

To describe our experience of reducing anastomotic leakage, a problem that has not been properly solved.

**Methods:**

Starting in January 2020, we began implementing our integrated strategy (application of an esophageal diameter-approximated slender gastric tube, preservation of the fibrous tissue around the residual esophagus and thyroid inferior pole anastomosis) in consecutive patients undergoing esophagectomy without a nasogastric tube or a nasal-jejunum feeding tube. Additionally, the blood supply at the site of the anastomosis was evaluated with a near-infrared fluorescence thoracoscope after the completion of esophagogastric anastomosis in the integrated strategy group.

**Results:**

Of 570 patients who were reviewed, 119 (20.9%) underwent the integrated strategy, and 451 (79.1%) underwent the conventional strategy. The rate of anastomotic leakage was 2.5% in the integrated strategy group and 10.2% in the conventional strategy group (p = 0.008). In the integrated strategy group, the site of most of the anastomotic blood supply was the residual esophagus dominant (82.4%), followed by the gastroesophageal dual-dominant (12.6%) and the gastric tube dominant (5.0%). The reconstruction route was more likely to be orthotopic in the integrated strategy group than in the conventional strategy group (89.9% vs. 38.6%, p = 0.004). Gastric dilation was identified in 3.4% of the patients in the integrated strategy group and in 21.1% in the conventional strategy group.

**Conclusions:**

Patients who underwent our proposed integrated strategy (Zhengzhou Strategy) during McKeown esophagectomy without a nasogastric tube or a nasal-jejunum feeding tube had a strikingly lower rate of anastomotic leakage and a relatively lower rate of postoperative complications, such as gastric tube dilation and delayed gastric emptying.

## 1 Introduction

Esophagogastric anastomosis has been widely discussed as it relates to esophagectomy and has undergone a constant evolution worldwide with improving perioperative recovery and survival outcomes. However, the problem of anastomotic leakage (AL) has not been properly solved. Among various potential risk factors for AL, poor perfusion/drainage and high tension at the anastomotic site are considered the most important. In the past half century, various types of gastric tubes have been described, aimed at reconstructing a well-perfused/drained or tension-free anastomosis. On the other hand, strategies other than gastric tube formation have been attempted, such as splenectomy, ischemic preconditioning, supercharged or/and superdrainage anastomosis, application of vasodilators (such as prostaglandin E1) and transient bloodletting, mainly aimed at improving the arterial blood supply or venous drainage. However, no optimal strategy has been widely endorsed, and most dogmas are based on the surgeon's personal experience and preferences.

In addition to AL, intrathoracic manifestations in cervical AL, gastric tube dilation, and delayed gastric emptying (DGE) are critical issues in patients who undergo esophagectomy. To solve the above issues that can be encountered during esophagectomy, we developed an integrated strategy (IS) in esophagogastric anastomosis to reduce esophagogastric AL, intrathoracic infection, gastric tube dilation, DGE and postoperative complications.

## 2 Patients and methods

### 2.1 Study population

Consecutive patients who underwent curative esophagectomy at our center between January 2017 and June 2022 were eligible for this study.

The inclusion criteria were patients aged ≥18 years, those with pathologically diagnosed esophageal squamous cell carcinoma (ESCC), and those who underwent the McKeown procedure with 2-field or 3-field lymph node (LN) dissection. A gastric tube passing through the posterior-mediastinal route was necessary.

The exclusion criteria were an inability to perform cervical esophagogastric anastomosis because of cervical esophagus cancer (short remnant esophagus) or history of gastric cancer (esophageal replacement with small or large intestine), palliative or salvage surgery, malignant tumor history or synchronous cancer. Severe comorbidities potentially causing serious perioperative complications were also excluded: Eastern Oncology Cooperative Group (ECOG) performance status of 2, ASA-physical status class ≥3 [1], disease of the cardiovascular system (ischemic heart disease, cardiac failure, uncontrolled hypertension), disease of the respiratory system (interstitial pneumonia, respiratory failure, uncontrolled bacterial or viral pneumonia), disease of the digestive system (liver cirrhosis, active hepatitis), disease of the urinary system (chronic renal failure requiring hemodialysis), disease of the endocrine system (uncontrolled diabetes mellitus) and other conditions (systemic infection requiring treatment, continuous administration of an immunosuppressant agent or steroid).

### 2.2 Surgical procedure

#### 2.2.1 Control procedure

All included patients with thoracic ESCC underwent thoracolaparoscopic radical esophagectomy with mediastinal and abdominal lymphadenectomy. The thoracic procedure was completed in the left lateral decubitus position, while the abdominal procedure and cervical anastomosis were completed in the supine position. After mobilization of the thoracic esophagus and abdominal stomach, a minilaparotomy incision of approximately 5 cm was made to complete gastric tube reconstruction. A 4 cm wide gastric tube was created using a linear stapler, and the cutting line was reinforced and embedded with 3-0 silk suture. Then, *trans*-postmediastinal pull-through of the gastric tube with a left-sided cervical end-to-side mechanical esophagogastric anastomosis was completed using a circular stapler. Anastomotic reinforcement was carried out. A nasogastric decompression tube (incisor distance 40 cm, distally near the level of the diaphragm hiatus) and nasal-jejunum feeding tube were routinely placed at the time of esophagectomy.

#### 2.2.2 Integrated strategy

**Fig. 1:**
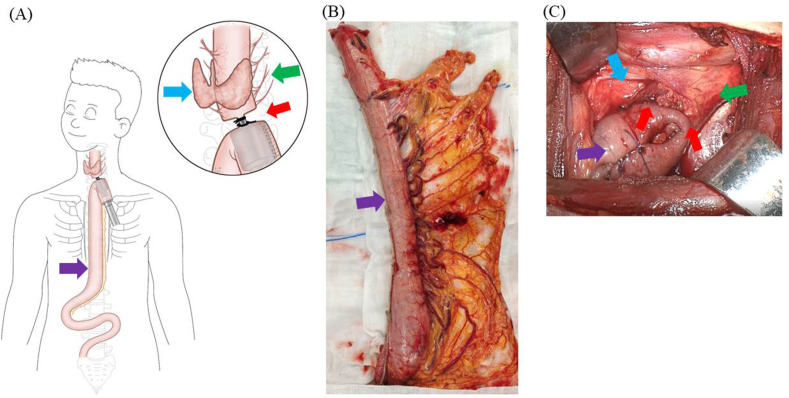
Our proposed IS in McKeown esophagectomy without nasogastric tube placement. (A) Schematic diagram of the IS. (B) Esophageal diameter-approximated slender gastric tube. (C) Cervical anastomosis. The solid blue arrow shows the thyroid; the solid green arrow shows the fibrous tissue (microvessels) around the residual esophagus; the solid red arrow shows the inferior pole anastomosis of the thyroid; the solid purple arrow shows the esophageal diameter-approximated slender gastric tube. IS, integrated strategy. (For interpretation of the references to colour in this figure legend, the reader is referred to the Web version of this article.)

The thoracic and abdominal procedures were the same as those in the control procedure. A slender gastric tube resembling the diameter of the esophagus was designed and reconstructed using a linear stapler to achieve a sufficient gastric tube length. Then, the cervical esophagus was mobilized to the inferior pole of the thyroid to ensure ultrahigh position esophagogastric anastomosis. There were bands of fibrous tissue between the cervical esophagus and the surrounding organs, and these fibrous tissues were protected properly during cervical esophagus mobilization to ensure that the esophagogastric anastomosis was well perfused by the residual esophagus and that the anastomosis was relatively fixed (“shorten the length of the ‘naked’ residual esophagus”, as we previously reported) [2]. Finally, end-to-side mechanical esophagogastric anastomosis near the inferior pole of the thyroid was performed using a circular stapler, and no anastomotic reinforcement was carried out. Additionally, no nasogastric tube or nasal-jejunum feeding tube was placed. Fig. 1 shows schematic drawings (Fig. 1A) and intraoperative photographs (Fig. 1B and. C) of the IS in esophagogastric anastomosis.

### 2.3 Postoperative management and follow-up

According to our protocol regarding postoperative care after esophagectomy, the endotracheal tube was routinely removed, and the patient was returned to the thoracic surgery ward. The bedside chest radiograph was completed on postoperative day (POD) 1 to evaluate the position and dilation of the gastric tube. Any unusual condition was further investigated by computed tomography (CT), and gastric decompression was implemented in patients with dilated tubes. Usually, the nasogastric decompression tube was removed on POD3 in the CS group, and oral feeding resumed on POD 7 in both groups. The nasal-jejunum feeding tubes were removed from the patients in the CS group on PODs 8–10, and the patients in both groups were subsequently discharged with instructions to maintain a semiliquid diet. The patients were switched to a normal diet on POD 14 and then regularly followed up.

### 2.4 Endpoints

The primary endpoint was the occurrence of AL. The secondary endpoints were blood supply at the anastomosis (IS group only), esophageal bed reconstruction, gastric tube dilation and DGE after esophagectomy. We also analyzed the occurrence of other postoperative complications: intrathoracic manifestations of cervical AL, cardiopulmonary complications (such as arrhythmia, pneumonia, atelectasis, hydrothorax, and chylothorax), and recurrent laryngeal nerve (RLN) injury.

AL was defined as symptomatic (inflammation cervical wound, digestive content spillover) or asymptomatic disruption of the cervical esophagogastric anastomosis. Intrathoracic manifestations included mediastinal abscess, mediastinitis or empyema thoracis. Regarding the reconstruction route, we classified the posterior-mediastinal route of the gastric tube into 2 subgroups: (A) the retrocardiac orthotopic route, which was placement of the gastric tube in the prevertebral esophageal bed (Fig. 3A), or (B) the intrathoracic heterotopic route, which was partial or total placement of the gastric tube in the thoracic cavity (Fig. 3B). Gastric tube dilation was classified as full gastric tube dilatation (Fig. 4A–D), proximal gastric tube dilatation (Fig. 5A), middle gastric tube dilatation (Fig. 5B) and distal gastric tube dilatation (Fig. 5C).

**Fig. 2:**
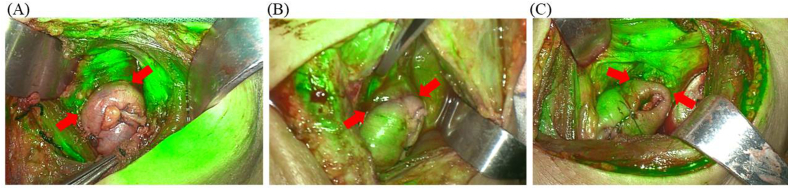
Blood supply at the anastomosis exhibited by indocyanine green-enhanced fluorescence. (A) Residual esophagus dominant; (B) Gastric tube dominant; (C) Gastroesophageal dual-dominant. The solid red arrow shows the esophagogastric anastomosis. (For interpretation of the references to colour in this figure legend, the reader is referred to the Web version of this article.)

In the IS group, the blood supply at the site of the anastomosis was evaluated with a near-infrared fluorescence thoracoscope (FLI-20A, Nanjing Nuoyuan Medical Devices Co., Ltd., Jiangsu, China) after the completion of esophagogastric anastomosis. Five milligrams of ICG was intravenously injected through the central line followed by a 10 ml flush of saline solution [3]. However, fluorescence quantification has several inevitable biases: 1) the time taken for the residual esophagus and gastric tube to become perfused are considerably different, 2) the distance between the fluorescent lens and tissue influences the judgment of perfusion and the measurement of fluorescence intensity, and 3) the patient's height, weight, heart rate, blood pressure and variation in the right gastroepiploic artery, which may have an impact on fluorescence intensity, can also introduce significant research bias. Thus, to ensure the unbiasedness of the result, images were captured at the time points when the absolute fluorescence intensity near the anastomosis on the residual esophagus or gastric tube was maximum. We only focused on the maximum fluorescence intensity near the anastomosis on the residual esophagus (denoted as N1) and gastric tube (denoted as N2) and then compared them with each other to determine the main source of blood supply. Three types of anastomotic blood supply sources were proposed: (A) residual esophagus dominant (N1/N2≥2) (Fig. 2A); (B) gastric tube dominant (N1/N2≤0.5) (Fig. 2B); and (C) gastroesophageal dual-dominant (0.5 < N1/N2<2) (Fig. 2C).

**Fig. 3:**
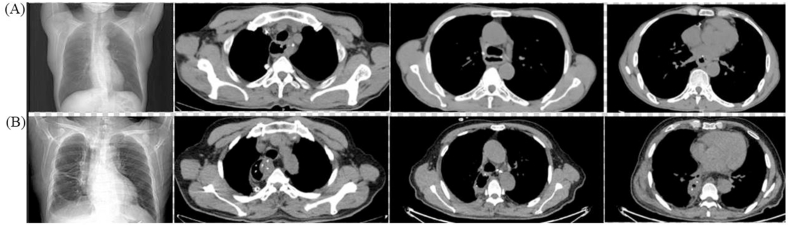
Posterior-mediastinal route of the gastric tube. (A) Retrocardiac orthotopic route (original esophageal bed); (B) Intrathoracic heterotopic route. From left to right: chest radiograph, level below the thoracic inlet, azygos arch level, inferior pulmonary vein level.

**Fig. 4:**
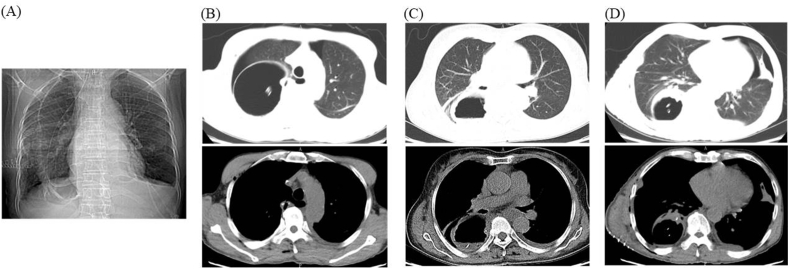
Full gastric tube dilatation. (A) Full gastric tube dilatation on DR; (B) proximal gastric tube dilatation; (C) middle gastric tube dilatation; (D) distal gastric tube dilatation.

**Fig. 5:**
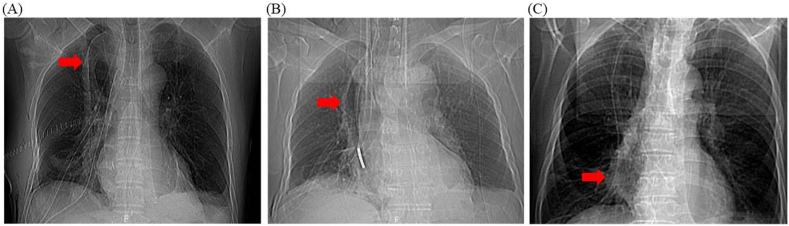
Classification of gastric tube dilatation. (A) Proximal gastric tube dilatation; (B) middle gastric tube dilatation; (C) distal gastric tube dilatation. The solid red arrow shows gastric tube dilatation. (For interpretation of the references to colour in this figure legend, the reader is referred to the Web version of this article.)

### 2.5 Statistical analysis

Prior to statistical analysis, the normality of continuous variables was tested using the Shapiro‒Wilk test. Continuous variables with a normal distribution are presented as the mean ± standard deviation (SD), and differences between them were evaluated using the *t*-test. Continuous variables with a skewed distribution are presented as the median (P25, P75), and differences between them were evaluated using the Wilcoxon rank-sum test. Categorical variables are expressed as frequencies (proportions), and differences between them were evaluated using the chi-square test or Fisher's exact test. The patients in the IS and CS groups were propensity score-matched (PSM) at a ratio of 1:2 with a match tolerance of 0.2 to adjust for baseline variables (age, sex, BMI, comorbidities, tumor location, cStage and neoadjuvant therapy). A p value of <0.05 was defined as statistically significant. IBM SPSS version 25 (SPSS Inc., Chicago, IL, USA) was used for data analysis.

## 3 Results

### 3.1 Patients

The IS was performed from January 2020 to June 2022, and the CS was performed from January 2017 to December 2019. Of 570 patients who were reviewed, 119 (20.9%) underwent IS, and 451 (79.1%) underwent CS. The characteristics are summarized in Table 1. There were no significant differences between the two groups, except for respiratory system comorbidities (p = 0.003) and neoadjuvant therapy regimen (0.046).

**Table 1:** Baseline characteristics of the included patients.

		Unmatched cohort			Matched cohort		
variable		integrated strategy (n = 119)	conventional strategy (n = 451)	P value	integrated strategy (n = 110)	conventional strategy (n = 220)	P value
Age	mean ± SD	64.6 ± 8.1	64.1 ± 7.5	0.463	64.3 ± 8.2	64.0 ± 7.8	0.751
Sex	male, n (%)	83 (69.7%)	318 (70.5%)	0.871	75 (68.2%)	150 (68.2%)	1.000
	female, n (%)	36 (30.3%)	133 (29.5%)		35 (31.8%)	70 (31.8%)	
BMI	kg/m^2^, mean ± SD	23.6 ± 3.0	23.6 ± 3.2	0.905	23.7 ± 3.0	23.6 ± 3.2	0.902
Comorbidities	Cardiovascular system^a^	35 (29.4%)	148 (32.8%)	0.479	32 (29.1%)	59 (26.8%)	0.663
	Respiratory system^b^	20 (16.8%)	35 (7.8%)	0.003	11 (10.0%)	23 (10.5%)	0.898
	Digestive system disease^c^	15 (12.6%)	37 (8.2%)	0.138	11 (10.0%)	25 (11.4%)	0.708
	Endocrine system disease^d^	9 (7.6%)	53 (11.8%)	0.192	9 (8.2%)	19 (8.6%)	0.889
Tumor location	Ut, n (%)	9 (7.6%)	64 (14.2%)	0.152	9 (8.2%)	26 (11.8%)	0.542
	Mt, n (%)	48 (40.3%)	174 (38.6%)		47 (42.7%)	85 (38.6%)	
	Lt, n (%)	62 (52.1%)	213 (47.2%)		54 (49.1%)	109 (49.5%)	
cStage	I, n (%)	21 (17.6%)	69 (15.3%)	0.212	20 (18.2%)	29 (13.2%)	0.303
	II, n (%)	43 (36.1%)	205 (45.5%)		39 (35.5%)	96 (43.6%)	
	III, n (%)	47 (39.5%)	160 (35.5)		44 (40.0%)	87 (39.5%)	
	IV, n (%)	8 (6.7%)	17 (3.8%)		7 (6.4%)	8 (3.6%)	
Neoadjuvant therapy	chemotherapy, n (%)	12 (10.1%)	56 (12.4%)	0.046	11 (10%)	27 (12.3%)	0.273
	chemotherapy + immunotherapy, n (%)	14 (11.8%)	22 (4.9%)		12 (10.9%)	12 (5.5%)	
	chemotherapy + Anlotinib, n (%)	0 (0%)	1 (0.2%)		0 (0%)	1 (0.5%)	

BMI, body mass index; Lt: lower thoracic esophagus; Mt: middle thoracic esophagus; SD, standard deviation; Ut, upper thoracic esophagus.

a includes arrhythmia, atrioventricular block, hypertension.

b includes COPD (FEV 1.0% < 70%), old pulmonary tuberculosis, bronchiectasis.

c includes hepatitis, intestinal polyp, hiatus hernia, gastric ulcer, gallbladder stone/post cholecystectomy, post appendectomy, inguinal hernia.

d includes hyperthyroidism, hypothyroidism, diabetes mellitus.

### 3.2 Postoperative outcomes and complications

As shown in Table 2, the total operation times for the IS and CS were 315 ± 87.4 min and 308 ± 64.6 min, respectively, which were not different among the groups (p = 0.446). The proportions of 3-field LN dissection were 6.7% (8/119) and 9.3% (42/451), respectively, and were not different among the groups (p = 0.374). The lengths of postoperative hospital stay were 12.0 ± 8.0 days and 14.0 ± 10.0 days for the IS and CS groups, respectively, which were significantly different (p = 0.027).

**Table 2:** Surgical outcomes and postoperative complications before and after PSM (1:2 ratio).

		Unmatched cohort			Matched cohort		
Variable		integrated strategy (n = 119)	conventional strategy (n = 451)	P value	integrated strategy (n = 110)	conventional strategy (n = 220)	P value
Total operation time	minutes, mean ± SD	315 ± 87.4	308 ± 64.6	0.446	318 ± 86.1	315 ± 66.5	0.724
LN dissection, n (%)	2-field, n (%)	111 (93.3%)	409 (90.7%)	0.374	102 (92.7%)	202 (91.8%)	0.773
	3-field, n (%)	8 (6.7%)	42 (9.3%)		8 (7.3%)	18 (8.2%)	
Anastomotic leakage	Cervical AL, n (%)	3 (2.5%)	46 (10.2%)	0.008	3 (2.7%)	19 (8.6%)	0.042
	Intrathoracic manifestation, n (%)	0/3 (0%)	21/46 (45.7%)	NA^a^	0/3 (0%)	8/19 (42.1%)	NA^a^
Blood supply at anastomosis	Residual esophagus dominant, n (%)	98 (82.4%)	NA	NA^a^	90 (81.8%)	NA	NA^a^
	Gastric tube dominant, n (%)	6 (5.0%)			6 (5.5%)		
	Gastro-esophageal dual-dominant, n (%)	15 (12.6%)			14 (12.7%)		
Reconstruction route	Esophageal bed, n (%)	107 (89.9%)	174 (38.6%)	<0.001	101 (91.8%)	88 (40.6%)	<0.001
	Intrathoracic, n (%)	12 (10.1%)	277 (61.4%)		9 (8.2%)	129 (59.4%)	
Gastric tube dilation	Total, n (%)	4 (3.4%)	95 (21.1%)	<0.001	2 (1.8%)	47 (21.4%)	<0.001
	Full gastric tube dilatation	0 (0%)	6 (6.3%)		0 (0%)	4 (1.8%)	
	Proximal gastric tube dilatation	4 (100%)	85 (89.5%)		2 (100%)	40 (18.2%)	
	Middle gastric tube dilatation	0 (0%)	2 (2.1%)		0 (0%)	2 (0.9%)	
	Distal gastric tube dilatation	0 (0%)	2 (2.1%)		0 (0%)	1 (0.5%)	
Delayed gastric emptying	n (%)	8 (6.7%)	94 (20.8%)	<0.001	6 (5.5%)	46 (21.2%)	<0.001
Cardiopulmonary complications	Arrhythmia, n (%)	8 (6.7%)	44 (9.8%)	0.307	7 (6.4%)	26 (12.0%)	0.111
	Pneumonia, n (%)	37 (31.1%)	135 (29.9%)	0.806	37 (30.9%)	66 (30.4%)	0.927
	Atelectasis, n (%)	24 (20.2%)	148 (32.8%)	0.008	24 (21.8%)	66 (30.4%)	0.100
	Hydrothorax, n (%)	18 (15.1%)	126 (27.9%)	0.004	18 (16.4%)	63 (29.0%)	0.012
	Chylothorax, n (%)	1 (0.8%)	6 (1.3%)	0.666	1 (0.9%)	4 (1.8%)	0.515
Recurrent nerve palsy	n (%)	5 (4.2%)	49 (10.9%)	0.027	5 (4.5%)	25 (11.4%)	0.042
Unplanned ICU admission	n (%)	9 (7.6%)	40 (8.9%)	0.651	9 (8.2%)	23 (10.5%)	0.511
Postoperative mortality	n (%)	0 (0%)	3 (0.67%)^b^	NA^a^	0 (0%)	3 (1.36%)^b^	NA^a^
Postoperative hospital stay	day, mean ± SD	12.0 ± 8.0	14.0 ± 10.0	0.027	12.1 ± 8.2	13.9 ± 10.4	0.114

ICU, intensive care unit; LN, lymph node; PSM, propensity score matching; SD, standard deviation.

a Because the anastomosis blood supply was not evaluated in any of the patients in the conventional strategy group and there was no intrathoracic manifestation or deaths in the integrated strategy group, we did not perform the statistical analysis.

b 2 died from cervical anastomotic fistula with intrathoracic manifestation, and 1 died from acute heart failure.

Regarding postoperative complications, patients in the IS group were less likely to be diagnosed with DGE (6.7% (8/119) vs. 20.8% (94/451), p < 0.001). The rates of arrhythmia, pneumonia, and chylothorax associated with IS and CS were 6.7% and 9.8% (p = 0.307), 31.1% and 29.9% (p = 0.806), and 0.8% and 1.3% (p = 0.666), respectively, without statistical significance. However, the rates of atelectasis (20.2% (24/119) vs. 32.8% (148/451), p = 0.008) and hydrothorax (15.1% (18/119) vs. 27.9% (126/451), p = 0.004) in the IS group were significantly lower than those in the CS group. The prevalence of an RLN injury was lower in the IS group than in the CS group (4.2% vs. 10.9%, respectively; p = 0.027). The rates of UIA were 7.6% and 8.9%, respectively (p = 0.651). No patients in the IS group died during the postoperative period, while 3 patients in the CS group died (2 died from cervical anastomotic fistula with intrathoracic infection, and 1 died from acute heart failure).

### 3.3 Indicators focusing on blood supply and tension at the anastomotic site

The rate of AL was 2.5% (3/119) in the IS group and 10.2% (46/451) in the CS group (p = 0.008). As shown in Supplementary Table 1, univariable and multivariable logistic regression analysis revealed that the integrated strategy was a protective factor for AL. In patients with cervical AL, there was no intrathoracic inflammation (0/3) in the IS group, and 45.7% (21/46) of patients in the CS group had intrathoracic inflammation.

In the IS group, most of the blood supply at the anastomosis site was residual esophagus dominant (82.4%), followed by gastro-esophageal dual-dominant (12.6%) and gastric tube dominant (5.0%). The reconstruction route was more likely to be at the original esophageal bed in the IS group than in the CS group (89.9% (107/119) vs. 38.6% (174/451), p = 0.004). Gastric dilation, assessed by chest X-ray on POD 1, requiring nasogastric tube adjustment was less common in the IS group, with 3.4% (4/119) of patients requiring this in the IS group and 21.1% (95/451) in the CS group. Furthermore, in patients with gastric dilation, proximal gastric tube dilatation was the most prevalent, with 100% (4/4) in the IS group and 89.5% (89/95) in the CS group.

To reduce the potential impact of variables on the results, we conducted PSM to adjust the baseline variables between the two groups. Two matched groups (110 pairs, n = 330 patients) were generated, among which there were no significant differences between the baseline clinical characteristics (Table 1). Postoperative DGE, hydrothorax rate, RLN injury prevalence, AL rate, anastomosis blood supply, reconstruction route and gastric dilation were consistent with the conclusion before PSM. However, there was no difference in the postoperative atelectasis rate or postoperative hospital stay between the two groups after PSM (Table 2).

## 4 Discussion

The impact of AL on postoperative recovery and quality of life is significant. In this study, we demonstrated the concept and method of constructing a gastric tube based on an IS; this IS appeared to be associated with reduced AL or other postoperative complications. Furthermore, potential mechanisms were explored.

Specifically, there are several important considerations (application of an esophageal diameter-approximated slender gastric tube, preservation of the fibrous tissue around the residual esophagus and inferior pole anastomosis of the thyroid) when implementing this IS, allowing the potential benefits, such as less gastric tube morbidity and postoperative complications, of this strategy to be realized.

The contribution of narrow and wide gastric tubes to AL is controversial. Wide gastric tubes, especially near the fundus of the stomach [4,5], may have the advantage of preserving the blood supply to the anastomosis. On the other hand, the lesser curvature often cannot be sufficiently released in a wide gastric tube to achieve a tension-free anastomosis [6]. Studies have attempted to integrate the strengths of narrow and wide gastric tubes with a sufficiently stretched gastric tube, potential storage function or properly preserved submucosal blood vessels of the gastric fundus [4,5,7,8]. However, does a more advanced vascular plexus necessarily mean a sufficient blood supply to the anastomosis? The vascular plexus at the gastric fundus is properly preserved in a “flexible” gastric tube and bat-shaped gastric tube, leading to a relatively low AL rate, as reported. However, there is also an opposite point of view: the reduced size of the stomach in a narrow gastric tube will lead to a relative increase in blood supply to the gastric tube. Correspondingly, a wider proximal gastric tube would render that area even more ischemic. A previous study [8] reported the use of a different coniform gastric tube, in which the technique has been demonstrated to destroy the vascular plexus in the center of the stomach. Indocyanine green-enhanced fluorescence was used to assess the perfusion of the anastomosis and revealed that the anastomosis was well perfused. Therefore, the question arises regarding the mechanism behind the association between the different shapes of gastric tubes and less AL. It might be possible that the destruction of the vascular plexus at the gastric fundus may be offset by a closer anastomosis near the end of the right gastroepiploic artery. Additionally, a wider gastric tube may cause iatrogenic injury (damage and subsequent ischemia under traction forces) during transmediastinal pull-through, suggesting that the bulky omentum should be removed to facilitate transmediastinal pull-through.

On the other hand, we noticed bands of fibrous tissue between the cervical esophagus and the surrounding organs and microvessels embedded in the fibrous tissue [2]. Can cervical anastomosis with maintained fibrous tissue (microvessels) between the esophagus and the surrounding organs improve perfusion at the anastomotic site? As shown in our data, in our IS group, the blood supply of most of the anastomoses was residual esophagus dominant (82.4%), indicating that the influence of the residual esophagus on the blood perfusion of an esophagogastric anastomosis cannot be neglected. However, we do not have the corresponding data for the CS group due to the retrospective nature of our study. There is still concern whether the tip of a slender gastric tube increases the risk of ischemia and the risk of leakage at the tip of the gastric tube rather than at the anastomotic site. However, our integrated strategy may have compensated for the ischemic condition at the tip of the gastric tube by preserving the fibrous tissue around the residual esophagus. Thus, the tip of the gastric tube may not be a critical clinical issue, at least in our integrated strategy.

Gastric tube dilatation is another nonnegligible factor for AL after esophagectomy. Dilatation of the gastric tube can occur for numerous reasons, such as wide gastric tube construction, out-of-position placement of the nasogastric tube and DGE. Measures have been taken to reduce the probability of gastric tube dilatation, such as narrow gastric tube construction, proper gastric tube decompression, pyloromyotomy [9], pleural flap application and esophageal bed reconstruction. There is mounting evidence that the gastric tube functions more as a conduit than as a reservoir and is unlikely to impact nutritional status. Gastric tube dilatation, as shown in our study, occurred significantly more frequently in the CS group, which proved that narrow gastric tube construction can reduce the rate of dilation to some degree because the narrow gastric tube is less likely to be patulous and distended. On the other hand, there are 2 potential reconstruction routes for transferring the gastric tube to the left neck: the retrocardiac orthotopic route (original esophageal bed) and the intrathoracic heterotopic route. The reconstruction route was more likely to be orthotopic in the IS group than in the CS group, which may further reduce the possibility of gastric tube dilatation. Furthermore, as shown in our study, patients in the IS group were less likely to be diagnosed with DGE, which can also be explained by original esophageal bed reconstruction. Regarding pulmonary complications, we found that the rates of hydrothorax in the IS group were significantly lower than those in the CS group. Thus, our reconstruction strategy might confer a significant advantage in terms of pulmonary complications. The underlying mechanism could be less lung compression by the gastric tube in patients with original esophageal bed reconstruction. More crucially, patients without a nasogastric tube or a nasal-jejunum feeding tube may have good cough and expectoration ability. Interestingly, in the opinion of some experts, jejunal graft conduits after esophagectomy are uniquely advantageous, partly because the jejunum has a luminal size similar to that of the esophagus, which also supports our proposed concept of an esophageal diameter-approximated slender gastric tube.

A substantial proportion (27%–62%) [[10], [11], [12]] of patients with cervical AL may exhibit potentially life-threatening intrathoracic manifestations, such as mediastinal abscesses, empyema thoracis and gastric tube-bronchial fistula. The rate of intrathoracic manifestations of AL was 45.7% in our CS group, which was consistent with previous studies. Correspondingly, AL with manifestations confined to the cervical region is not serious or fatal. Strategies to confine the AL to the cervical region have been explored, such as pleural flaps or greater omentum application. In our IS group, we put forward the concept of “thyroid inferior pole anastomosis”, along with shortening of the length of the “naked” residual esophagus. The anastomosis was fixed and located at a relatively high position. As shown in Table 2, we reported a 0% intrathoracic manifestation in the IS group with 3 cervical ALs, which suggested that incorporation of this IS has the potential to prevent life-threatening intrathoracic manifestations. Moreover, a nasogastric tube and a nasal-jejunum feeding tube were placed in the CS group but not in the IS group, which is a noteworthy aspect of the IS. Generally, gastric tube dilation poses a significant problem in the implementation of a tube-free strategy. However, the dilation rate in the implementation of our strategy was 3.4%. The advantage of our strategy was our relatively slender gastric tube. Additionally, the most likely site of reconstruction involved the original esophageal bed in the IS group. Considering the increased use of enhanced recovery after surgery (ERAS) strategies following esophagectomy, an increasing number of centers have started implementing a tube-free strategy without affecting patients’ functional recovery or the rate of severe postoperative complications such as AL [13,14]. Our study confirms that the tube-free strategy is safe to some extent.

## 5 Conclusion

An IS for intraoperative implementation in McKeown esophagectomy without a nasogastric tube or a nasal-jejunum feeding tube was introduced to reduce AL. We found a strikingly lower rate of AL and a relatively lower rate of postoperative complications, such as gastric tube dilation and DGE, which suggested that the application of an esophageal diameter-approximated slender gastric tube, preservation of the fibrous tissue (microvessels) around the residual esophagus and inferior pole anastomosis of the thyroid contributed to the improvement in the esophagectomy results. Further studies are needed to establish this IS as the standard of care.

## Declarations

This study was reviewed and approved by the Zhengzhou University Institutional Review Board (No.: 2022-KY-1240-001 ), and the requirement for informed consent was waived because our study was retrospective.

## Data availability statement

Data will be made available on request.

## Funding

This study was supported by the Key Scientific Research Projects of the Institutions of Higher Learning in Henan Province (No. 21A320032)

## CRediT authorship contribution statement

**Yan Zhang:** Writing – review & editing, Writing – original draft, Visualization, Validation, Software, Resources, Project administration, Methodology, Investigation, Formal analysis, Data curation, Conceptualization. **Junya Wang:** Writing – review & editing, Writing – original draft, Visualization, Validation, Software, Resources, Project administration, Methodology, Investigation, Formal analysis, Data curation, Conceptualization. **Shuang Ren:** Writing – review & editing, Writing – original draft, Visualization, Validation, Software, Resources, Project administration, Methodology, Investigation, Formal analysis, Data curation, Conceptualization. **Jia Jiao:** Writing – original draft, Visualization, Validation, Software, Resources, Project administration, Methodology, Investigation, Formal analysis, Data curation, Conceptualization. **Zheng Ding:** Writing – original draft, Validation, Software, Resources, Methodology, Conceptualization. **Hang Yang:** Writing – original draft, Software, Resources, Methodology. **Dabo Pan:** Writing – original draft, Software, Resources, Methodology. **Jindong Li:** Writing – review & editing, Writing – original draft, Visualization, Validation, Supervision, Software, Resources, Project administration, Methodology, Investigation, Formal analysis, Data curation, Conceptualization. **Guoqing Zhang:** Writing – review & editing, Writing – original draft, Visualization, Validation, Supervision, Software, Resources, Project administration, Methodology, Investigation, Formal analysis, Data curation, Conceptualization. **Xiangnan Li:** Writing – review & editing, Writing – original draft, Visualization, Validation, Supervision, Software, Resources, Project administration, Methodology, Investigation, Funding acquisition, Formal analysis, Data curation, Conceptualization. **Song Zhao:** Writing – review & editing, Writing – original draft, Visualization, Validation, Supervision, Software, Resources, Project administration, Methodology, Investigation, Formal analysis, Data curation, Conceptualization.

## Declaration of competing interest

The authors declare that they have no known competing financial interests or personal relationships that could have appeared to influence the work reported in this paper.
