# Supplementary material for: An integrated strategy for reducing anastomotic leakage in patients undergoing McKeown esophagectomy
Source: Heliyon. 2024 Feb 15;10(4):e26430. doi: 10.1016/j.heliyon.2024.e26430 (PMC10884487; doi:10.1016/j.heliyon.2024.e26430)
Supplement: Multimedia component 1 [file mmc1.docx]

Supplementary Table 1. Univariable and multivariable logistic regression analysis of risk factors for anastomotic leakage

|  |  | **Univariable analysis** |  | **multivariable** |  |
| --- | --- | --- | --- | --- | --- |
| **variable** |  | **OR (95% CI)** | **P value** | **OR (95% CI)** | **P value** |
| **Age** |  | 1.010(0.972-1.049) | 0.622 | 1.013(0.973-1.054) | 0.535 |
| **Sex** | male | baseline |  | baseline |  |
|  | female | 0.951(0.504-1.797) | 0.877 | 0.877(0.439-1.749) | 0.709 |
| **Hb** |  | 1.001(0.993-1.008) | 0.846 | 1.001(0.994-1.007) | 0.826 |
| **BMI** |  | 1.033(0.938-1.138) | 0.512 | 1.030(0.933-1.136) | 0.558 |
| **Tumor location** | Ut | baseline | 0.145 | baseline | 0.224 |
|  | Mt | 2.358(0.999-5.568) | 0.050 | 2.181(0.902-5.277) | 0.084 |
|  | Lt | 1.587(0.725-3.475) | 0.248 | 1.605(0.696-3.698) | 0.267 |
| **cStage** | I | baseline | 0.219 | baseline | 0.260 |
|  | II | 0.734(0.288-1.872) | 0.517 | 0.843(0.319-2.224) | 0.730 |
|  | III | 0.853(0.322-2.255) | 0.748 | 0.879(0.325-2.380) | 0.800 |
|  | IV | 0.286(0.079-1.031) | 0.056 | 0.291(0.075-1.127) | 0.074 |
| **Total operation time** |  | 0.997(0.993-1.001) | 0.157 | 0.997(0.993-1.001) | 0.174 |
| **Neoadjuvant therapy** | yes* | baseline |  | baseline |  |
|  | no | 1.150(0.554-2.385) | 0.708 | 1.177(0.547-2.534) | 0.677 |
| **Surgical procedure** | conventional strategy | baseline |  | baseline |  |
|  | integrated strategy | 0.228(0.070-0.745) | 0.014 | 0.233(0.070-0.777) | 0.018 |

* includes neoadjuvant chemotherapy, neoadjuvant immunotherapy;

BMI, body mass index; CI, confidence interval; Lt: lower thoracic esophagus; Mt: middle thoracic esophagus; Ut, upper thoracic esophagus.
